# Hyperthermic intracavitary nanoaerosol therapy (HINAT) as an improved approach for pressurised intraperitoneal aerosol chemotherapy (PIPAC): Technical description, experimental validation and first proof of concept

**DOI:** 10.3762/bjnano.8.272

**Published:** 2017-12-18

**Authors:** Daniel Göhler, Stephan Große, Alexander Bellendorf, Thomas Albert Falkenstein, Mehdi Ouaissi, Jürgen Zieren, Michael Stintz, Urs Giger-Pabst

**Affiliations:** 1 Research Group Mechanical Process Engineering, Institute of Process Engineering and Environmental Technology, Technische Universität Dresden, Münchner Platz 3, D-01062 Dresden, Germany; 2 Technologieorientierte Partikel-, Analysen- und Sensortechnik, Topas GmbH, Oskar-Röder Straße 12, D-01237 Dresden, Germany; 3 Clinic for Nuclear Medicine, University Hospital Essen, Hufelandstraße 55, D-45147 Essen, Germany; 4 Basic Research Laboratory Department of Surgery, St. Mary’s Hospital Herne, Ruhr University Bochum, Hölkeskampring 40, D-44625 Herne, Germany; 5 Department of Digestive and Oncologic Surgery, Colorectal Surgery Unit, Trousseau Hospital, Avenue de la République, F-33170 Chambray-lès Tours, France; 6 Department of General Surgery & Therapy Center for Peritoneal Carcinomatosis, St. Mary’s Hospital Herne, Ruhr University Bochum, Hölkeskampring 40, D-44625 Herne, Germany

**Keywords:** HINAT, intracavitary, intraperitoneal, nanoaerosol, PIPAC, pressurized, therapy

## Abstract

**Background:** The delivery of aerosolised chemotherapeutic substances into pressurised capnoperitonea has been reported to be more effective than conventional liquid chemotherapy for the treatment of peritoneal carcinomatosis. However, recent reports reveal limitations of the currently available technology.

**Material and Methods:** A novel approach for pressurised intraperitoneal aerosol chemotherapy (PIPAC), called hyperthermic intracavitary nanoaerosol therapy (HINAT), based on extracavitary generation of hyperthermic and unipolar charged aerosols, was developed. The aerosol size distribution, the spatial drug distribution and in-tissue depth penetration of HINAT were studied by laser diffraction spectrometry, differential electrical mobility analysis, time of flight spectrometry, scintigraphic peritoneography and fluorescence microscopy. All experiments were performed contemporaneous with conventional PIPAC for the purpose of comparison. Furthermore, a first proof of concept was simulated in anesthetised German Landrace pigs.

**Results:** HINAT provides a nanometre-sized (63 nm) unipolar-charged hyperthermic (41 °C) drug aerosol for quasi uniform drug deposition over the whole peritoneum with significantly deeper drug penetration than that offered by conventional PIPAC.

## Introduction

Reliable epidemiologic data concerning the dissemination of peritoneal carcinomatosis (PC) are not available, but it is estimated that this stage of disease is diagnosed yearly for about 20,000 patients in Germany [1]. Although various treatment approaches have been developed in recent decades, PC is, in most cases, still the ultimate cause of death once it is diagnosed. Similar to the treatment of other types of cancer, PC treatment suffers on an accurate and efficient application of active substances to affected regions.

In order to treat PC with adequately high doses of chemotherapeutic substances at minimal systemic toxicity, first approaches for locoregional/intracavitary chemotherapy (ICC) based on liquid drug instillation (LICC) were already developed about forty years ago [2–4]. Unfortunately, the effectivity of LICC is limited by low in-tissue penetration and the difficulties to distribute the drug-containing solution homogeneously throughout the tumoral tissue [5].

One promising and upcoming approach to treat end-stage patients suffering from enhanced PC is the Pressurised IntraPeritoneal Aerosol Chemotherapy (PIPAC), where the drug-containing solution is aerosolised within a pressurised carbon-dioxide-based capnoperitoneum (i.e., with a carbon-dioxide-inflated abdominal cavity) by means of a microinjection pump (MIP^®^) in combination with a high-pressure injector. The gas-like propagation behaviour of the aerosol and the overpressure (*p*_C_ = 12 mmHg = 1.6 kPa) within the capnoperitoneum are associated with a more homogeneous drug distribution [6–8] and a deeper in-tissue drug penetration [6–7] as provided by LICC. Thus, PIPAC has gained rapid acceptance for the treatment of PC in the last years.

However, recently published data obtained by ex vivo and postmortem animal analyses showed that the spatial drug distribution over the whole abdominal cavity based on the currently available PIPAC-MIP technology is not as homogeneous as expected [9–14]. In-tissue depth penetration analyses [9–10] as well as scintigraphic peritoneography [14] showed local hotspots in the opposite side of the nozzle outlet of the MIP^®^ and undersupplied regions such as areas lateral to the aerosol jet. The inhomogeneity in the drug deposition is attributed to the droplet size distribution and the droplet kinetic of the aerosol generated by the MIP^®^ [13]. Granulometric analyses of the MIP^®^ aerosol by laser diffraction spectrometry showed that around 97.5 vol % of the aerosol is composed of droplets between 3–200 µm with a modal volume-weighted droplet size of ≈25 µm. Such droplets are deposited immediately on the opposite size of the nozzle outlet due to gravitational settling and inertial impaction. Thus, Göhler et al. [13] concluded that the fraction of coarse droplets based on the current PIPAC-MIP technology is too large to provide a homogenous drug distribution.

The opportunities to optimize single-fluid nozzles (like the MIP^®^) required aerosol characteristics are severely restricted by physical circumstances and thus only minor improvements are expected. Consequently, we developed an improved PIPAC approach for the treatment of enhanced PC, called hyperthermic intracavitary nanoaerosol therapy (HINAT) that enables an even more homogeneous intracavitary drug application as well as deeper in-tissue drug penetration in conjunction with intracavitary hyperthermia. This article aims to present the HINAT approach together with a first proof of concept in direct comparison with MIP^®^-based PIPAC (designated as PIPAC-MIP).

## Description of the Hyperthermic Intracavitary Nanoaerosol Therapy (HINAT)

**Figure 1:**
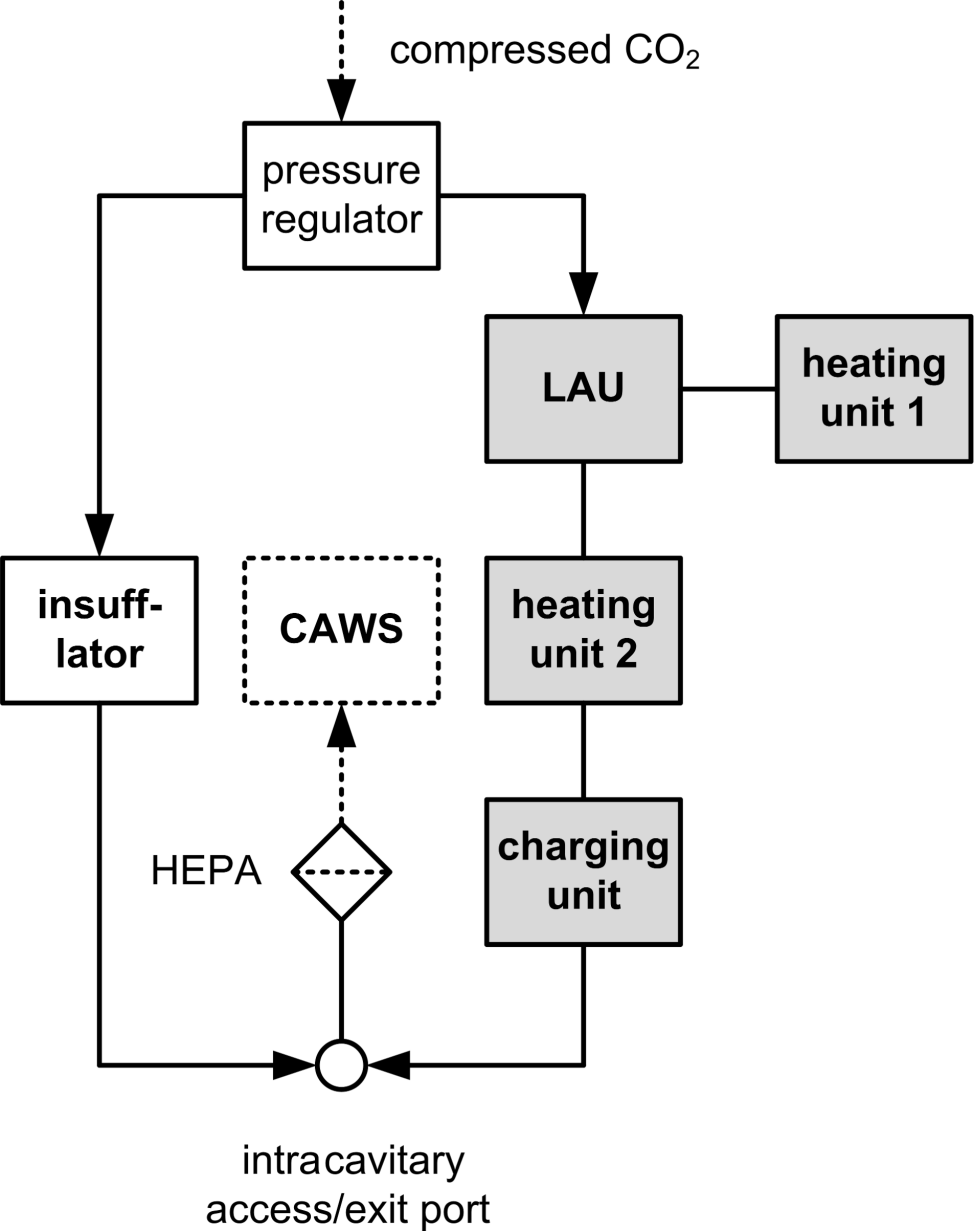
Schematic illustration of the HINAT approach; CAWS = closed air waste system, HEPA = high efficiency particulate air filter.

The HINAT approach, which is currently under patent application (DE 10 2016 202 316 A1 [15]), comprises improvements in aerosol generation and aerosol conditioning for the purpose of a more efficient drug application during PIPAC. A schematic illustration of the HINAT approach is given in Figure 1.

In contrast to PIPAC-MIP, the HINAT approach is based on extracavitary aerosol generation by means of a heatable liquid atomization unit (LAU). After generation, the aerosol passes a secondary heating unit and a charging unit before it is supplied into the abdominal cavity through an intracavitary access port (e.g., trocar or Veres needle).

### Extracavitary droplet generation

**Figure 2:**
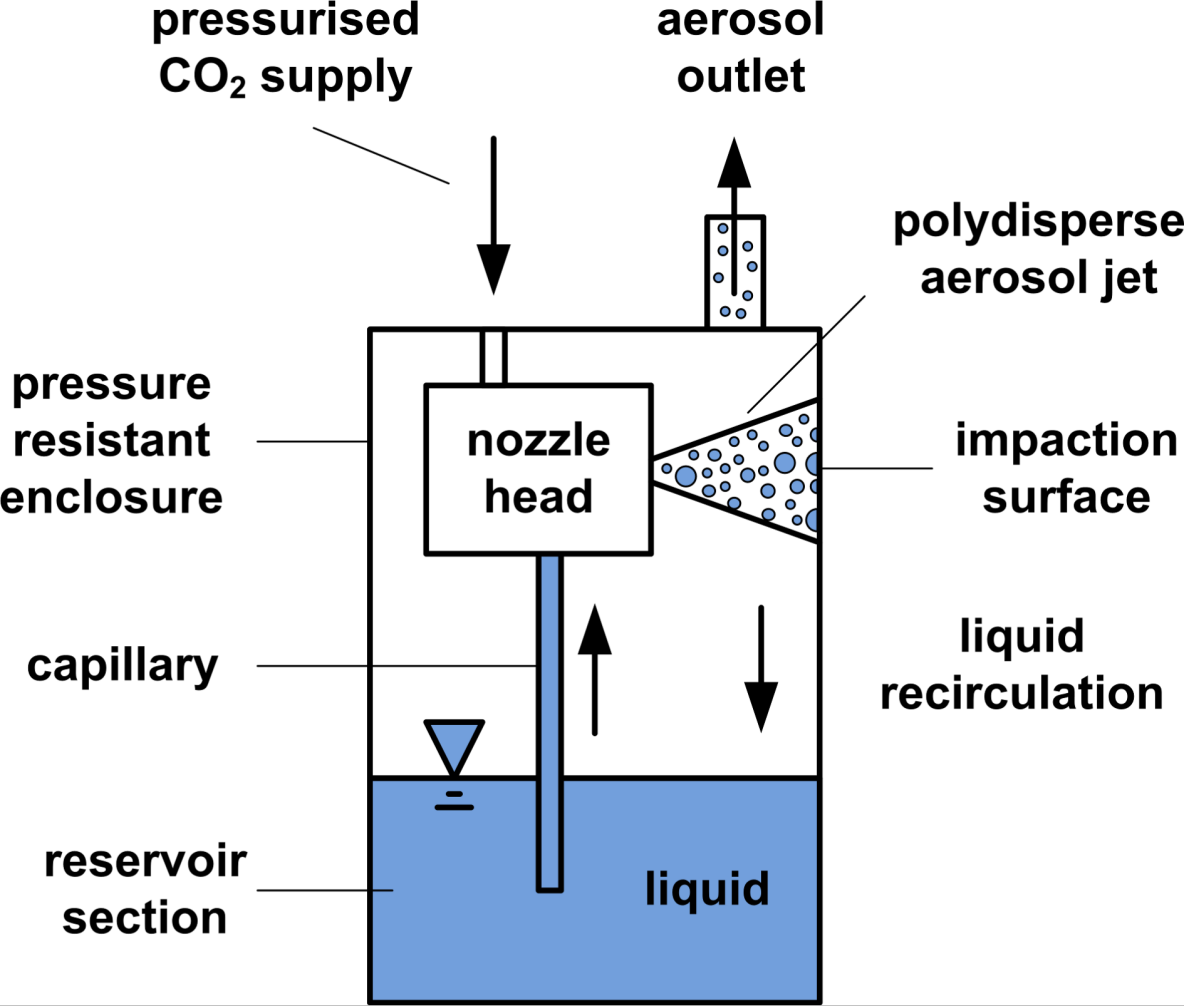
Schematic illustration of the extracavitary liquid atomisation unit (LAU).

The LAU illustrated in Figure 2 is operated with the same compressed carbon dioxide source as that is used for the establishment of the capnoperitoneum (*p*_C_ = 12 mmHg = 1.6 kPa). The aerosolisation takes place within a two-component nozzle located in the nozzle head, wherein the volumetric flow rate of pressurised carbon dioxide leads to a negative pressure in the capillary. The lower end of the capillary is partly submerged in the liquid drug to be aerosolised. The negative pressure causes a liquid flow rate into the nozzle head, where the carbon dioxide flow leads to shear-stress-induced droplet formation as well as droplet acceleration. The generated polydisperse aerosol leaves the nozzle head within a jet stream, which is directed perpendicular to the enclosure surface. The enclosure surface serves thus as an impaction plate to separate micrometre-sized droplets from submicrometre-sized ones. Impacted droplets flow as a liquid film back to the liquid reservoir, whereas submicrometre-sized droplets propagate within the chamber and leave the LAU through the aerosol outlet.

Preliminary analysis concerning the ejection performance of the LAU (injection nozzle diameter of 0.5 mm) based on compressed air showed that the volumetric aerosol flow rate as well as the liquid mass flow rate increase linearly with increasing system pressure for aqueous solutions. At a liquid temperate of 20 °C, the LAU provides a volumetric aerosol flow rate of 210 L/h and a liquid mass flow rate of 4.25 g/h at 1 bar system pressure, whereas a volumetric aerosol flow rate of 500 L/h and a liquid mass flow rate of 4.5 g/h is offered at a system pressure of 3 bar.

### Extracavitary aerosol conditioning (standard operation mode)

In order to use the benefits of hyperthermia (e.g., thermal cytotoxicity, increased drug penetration due to reduced intratumoral pressure, increased drug deposition due to improved thermophoretic conditions), both the liquid reservoir of the LAU as well as the aerosol that exits the LAU can be heated in a controlled manner to reach a defined aerosol temperature between 41 and 43 °C. Heating unit 1 is a thermostat, while heating unit 2 is a heating tube. Note that in the case of operating heating unit 1 (used for heating the liquid to be aerosolised), the mass flow rate increases significantly with increasing liquid temperature due to decreasing dynamic viscosity, density and surface tension of the liquid.

To increase the droplet flux as well as the in-tissue drug penetration homogenously over the whole peritoneum, the HINAT-LAU aerosol is also unipolar-charged before entering the capnoperitoneum. For this purpose, an approved brush electrode (Ionwand^TM^, Alesi Surgical Ltd, Cardiff, UK) is used in the setup to produce ionized gas molecules. Once the unipolar-charged aerosol comes close to the peritoneum, image charges are induced at the peritoneum that result in intensified attractive forces and thus to an improved droplet flux to the peritoneum.

### Intracavitary aerosol conditioning (optional operation mode)

To enable local, accelerated droplet deposition on the peritoneum, aerosol charging can also be performed intraperitoneal (cf. [8]) that requires an additional access into the abdominal cavity. Similar to the extracavitary charging, the supplied aerosol droplets are unipolar-charged by means of an approved brush electrode (Ionwand^TM^, Alesi Surgical Ltd, Cardiff, UK). In contrast to extracavitary charging, particle deposition during intracavitary charging is supported by the formation of an electrical field between electrode and grounded peritoneum. Due to the asymmetric conditions of the capnoperitoneum, peritoneal regions located in the near field of the electrode experience a more intensified droplet deposition than regions in the far field.

### Excess aerosol

In contrast to PIPAC-MIP, the HINAT-LAU aerosol is propagated with a considerably higher volumetric aerosol flow rate. To avoid a continuous pressure increase within the abdominal cavity, the supplied volumetric aerosol flow rate must be removed continuously. This can be performed through the same port as that is used for access or through a second displaced port. Depending on the actual volumetric aerosol flow rate and the corresponding fluid dynamic conditions, only a partial intraperitoneal drug deposition can be expected. Thus, remaining droplets of the excess aerosol have to be separated carefully from the gas with a HEPA filter before entering a closed air waste system (CAWS).

## Experimental

### Granulometric analyses (ex vivo)

#### Laser diffraction spectrometry (LDS)

Laser diffraction spectrometry (LDS) by means of a He–Ne laser diffraction spectrometer [16] according to ISO 13320:2009 [17] (HELOS/KR-H2487, Sympatec GmbH, Germany) was performed to characterise the volume-weighted size distribution of the HINAT-LAU aerosol in a size range of 0.5–175 µm (focal distance of 100 mm). For the purpose of comparison, LDS analyses were performed similar to the ones for PIPAC-MIP aerosol characterisation by Göhler et al. [13]. Thus, the LAU was operated with the same aqueous glucose solution (Glucosterile^®^ 5%, Fresenius Kabi GmbH, Germany) at a similar distance of ≤5 mm between aerosol outlet and laser beam (laser wavelength of 632.8 nm) and with equal signal evaluation (i.e., Fraunhofer theory). The LAU was operated with compressed air at system pressures of 1 bar, 2 bar and 3 bar. The measurement time was set to 3 s and measurements were taken 5 times for each configuration.

#### Differential electrical mobility analyses (DEMA) and time-of-flight spectrometry (TOF)

**Figure 3:**
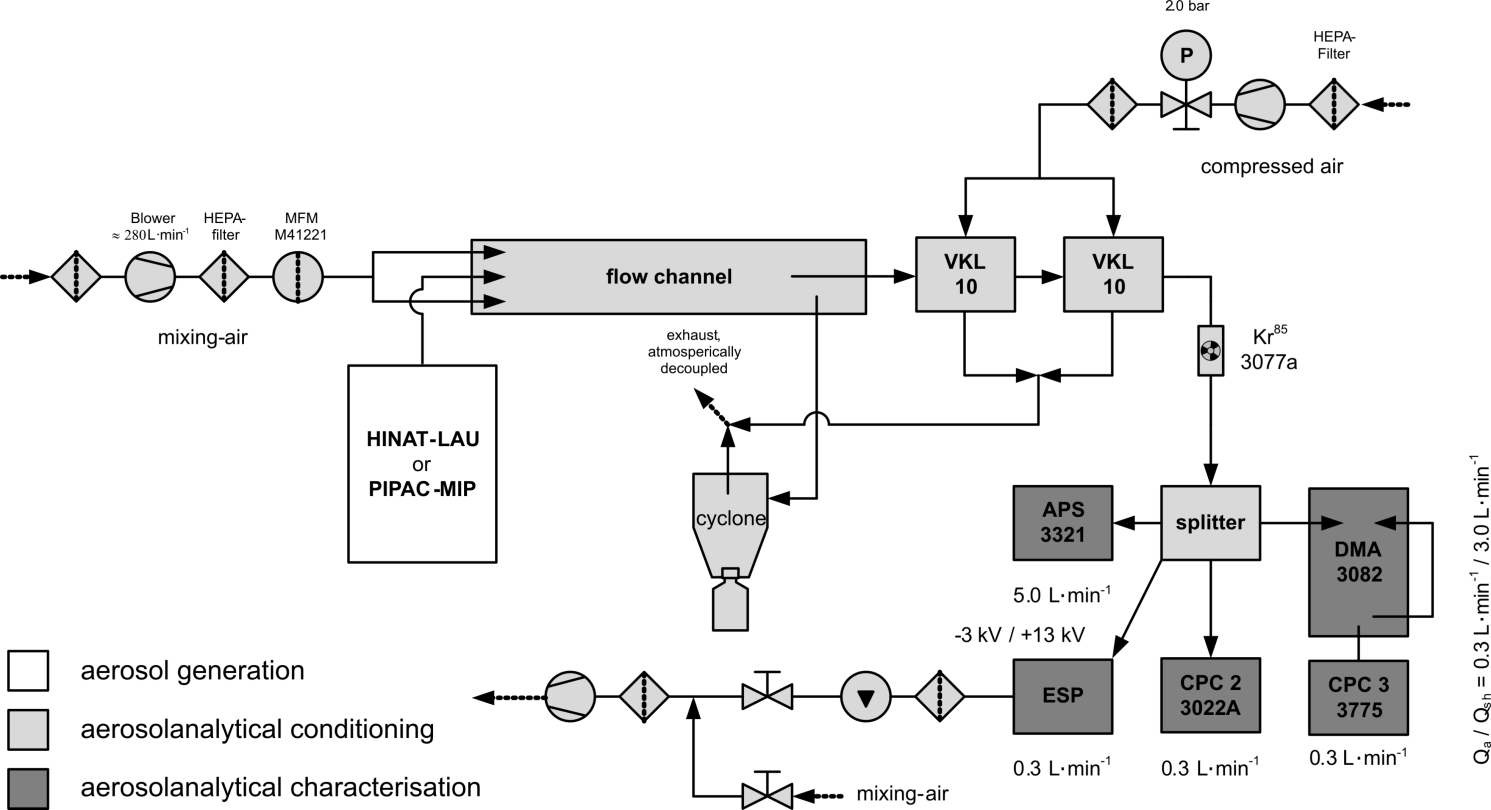
Schematic diagram of the operational experimental setup for the characterisation of the aerosols generated by HINAT-LAU and PIPAC-MIP.

Beside LDS, the aerosols provided by HINAT-LAU and PIPAC-MIP were also characterised down to a few nanometres by means of highly sensitive aerosol-analytic instruments. To ensure quantitative data evaluation, these analyses were performed within a flow channel. A schematic diagram of the operated experimental setup is given in Figure 3.

For the purpose of defined aerosol dilution, the aerosols generated from HINAT-LAU or PIPAC-MIP were passed parallel to a particle-free air flow of about 280 L/min into an atmospheric decoupled flow channel before entering a cascade of two commercial aerosol dilution units (model VKL10, Palas GmbH, Karlsruhe, Germany). Afterwards the aerosol passed a bipolar neutraliser (model 3077A, TSI Inc., Shoreview, USA) to improve the charge conditions for analyses. The operated aerosol-analytic instruments received the aerosol samples through a flow splitter. A scanning mobility particle sizing system (SMPS, model 3938, TSI Inc., Shoreview, USA) for differential electrical mobility analysis according to ISO 15900:2009 [18], composed of a bipolar neutralizer (model 3077a, TSI Inc., Shoreview, USA), a differential electrical mobility analyser (DMA, model 3082, TSI Inc., Shoreview, USA) and a condensation particle counter (CPC3, model 3775, TSI Inc., Shoreview, USA), was operated for the determination of the number-weighted particle size distribution and particle number concentration from 14–723 nm. A time-of-flight spectrometer (APS, model 3321, TSI Inc., Shoreview, USA) was employed for the characterisation of the number-weighted droplet size distribution and the particle number concentration between 0.5–20 µm. Furthermore, an additional condensation particle counter (CPC2, model 3022A, TSI Inc., Shoreview, USA) was used to determine the particle number concentration between 0.006 and 10 µm.

Both HINAT-LAU and PIPAC-MIP were operated with an aqueous glucose solution (Glucosterile^®^ 5%, Fresenius Kabi GmbH, Germany) at 28 °C. Cross-check analyses concerning possible vaporization effects within the experimental setup were performed with silicon oil (Elbesil-Oil B10, Böwing GmbH, Germany). The HINAT-LAU aerosol was characterised for a 1 bar and a 3 bar system pressure based on compressed air, while the MIP was operated in combination with a high pressure injector (Injektron 82 M, MedTron, Saarbrücken, Germany) at the standard liquid flow rate of 30 mL/min.

### Local drug deposition and in-tissue penetration in postmortem swine model

In order to characterise the effectivity of local drug deposition and in-tissue drug penetration, exposure experiments by HINAT-LAU and PIPAC-MIP were performed in postmortem German Landrace pigs (35–40 kg). The pigs were previously euthanized for a laparoscopic surgery training course. Both the training course as well as the experiments were approved by local authorities and the local board on animal welfare. All applicable international, national, and/or institutional guidelines for the use, handling and disposal of animal cadavers, cytostatic and radioactive materials were followed.

**Figure 4:**
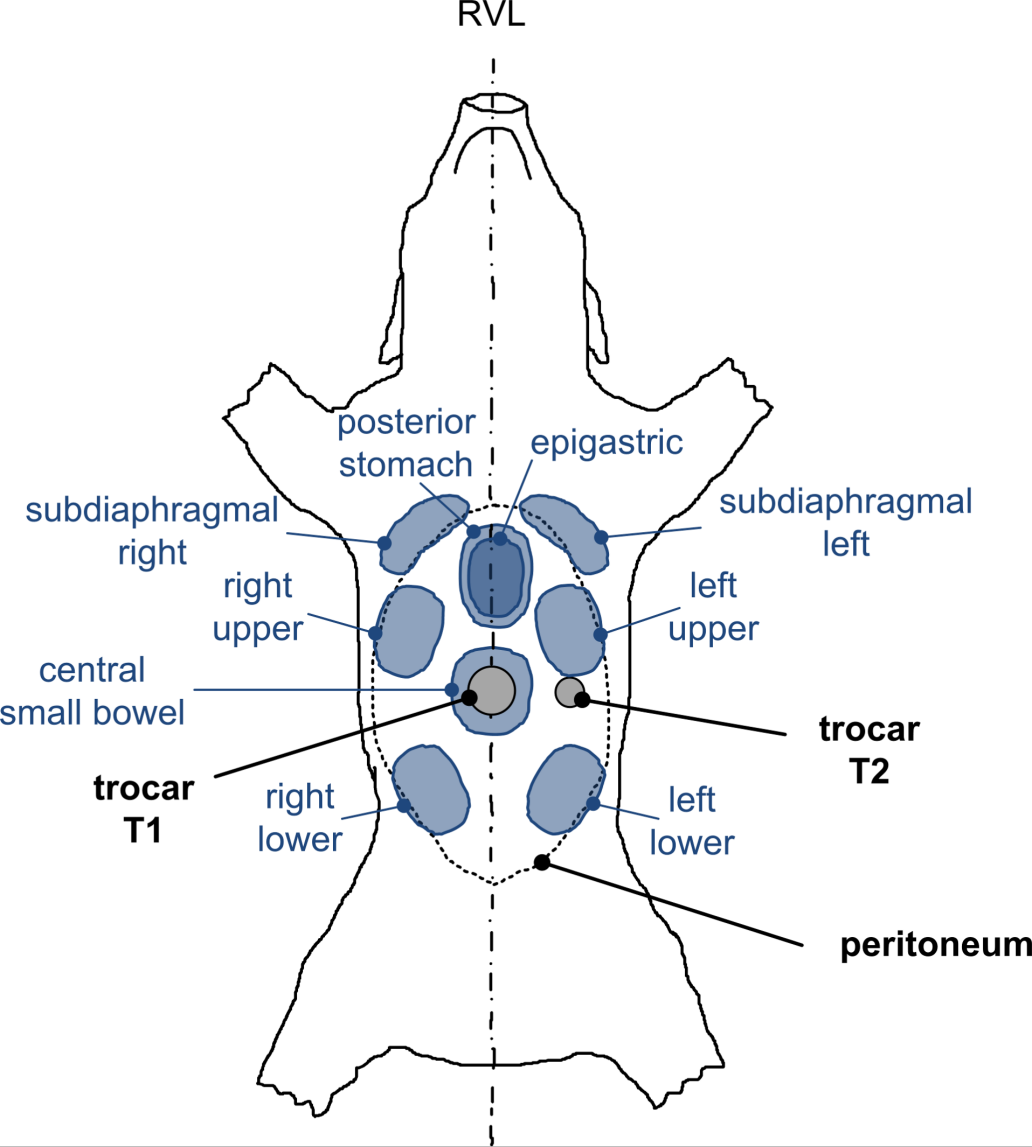
Sketch of pig cadaver with trocar positions and sampling regions for in-tissue penetration depth analyses; RVL = right–ventral–left.

The fresh pig cadavers were placed and fixed at all four extremities in a stable supine position similar to the sketch given in Figure 4. An infraumbilical minilaparotomy was performed to insert trocar T1 (Kii^®^Balloon Blunt Tip System, 12 mm, Applied Medical, Rancho Santa Margarita, CA, USA) into the abdominal cavity (peritoneal temperature of 28 °C due to postmortem cooling). Trocar T1 serves firstly to establish a capnoperitoneum (*p*_C_ = 12 mmHg = 1.6 kPa) that was maintained constant over time by means of an insufflator (UHI-3, Olympus medical life science and industrial divisions, Olympus Australia, Notting Hill, Australia). Afterwards a video endoscope (5 mm 30°, Karl Storz GmbH & Co KG, Tuttlingen, Germany) was fed through trocar T1 for the visual control of a second minilaparotomy at the left lateral-hemi abdomen, where trocar T2 (Kii^®^Balloon Blunt Tip System, 5 mm, Applied Medical, Rancho Santa Margarita, CA, USA) was inserted. Finally, the video endoscope was removed from trocar T1 and fed into trocar T2.

Both PIPAC application procedures (i.e., HINAT-LAU as well PIPAC-MIP) were performed via trocar T1 using separate pig cadavers. All PIPAC procedures (i.e., HINAT-LAU and PIPAC-MIP) were performed by a senior surgeon (GPU) experienced in PIPAC technology. Prior to aerosol application, the pig capnoperitonea were checked concerning tightness. After application, the capnoperitonea were evacuated through a high-efficiency particulate air (HEPA) filter system.

HINAT-LAU analyses were performed with an unheated aerosol based on compressed air that was also unipolar-charged extracavitary by means of a brush electrode (Ionwand^TM^, Alesi Surgical Ltd, Cardiff, UK) of an approved electrostatic precipitation system (Ultravision^TM^, Alesi Surgical Ltd, Cardiff, UK). The mentioned parameters were chosen in order to be as close as possible to those realised during granulometric analysis.

The PIPAC-MIP procedure was performed using a MIP^®^ (Reger Medizintechnik, Rottweil, Germany) connected by a high-pressure injection line (male/female Luer lock, 120 cm, 1200 psi, Smith Medical, Hranice, Czech Republic) with a high-pressure injector (Injektron 82 M, MedTron, Saarbrücken, Germany). The MIP^®^ was fed into trocar T1 and operated perpendicular with maximum distance to the small bowel serosal surface.

### Scintigraphic peritoneography (SPG)

Scintigraphic peritoneography (SPG) was performed after ^99m^Tc pertechnetate exposure of two postmortem pig peritonea by HINAT-LAU and PIPAC-MIP. An aqueous 0.9 wt % sodium chloride solution containing ^99m^Tc pertechnetate was aerosolised (activity concentration of 1 MBq/mL). The aerosol generation phase for both HINAT-LAU and PIPAC-MIP lasted 5 min. HINAT-LAU was performed with a system pressure of 1.4 bar that led to an effective dose of the ^99m^Tc pertechnetate-containing solution of 0.28 mL. PIPAC-MIP was performed at a volumetric flow rate of 30 mL/min for 5 min that led to a dose of 150 mL.

Short after ^99m^Tc pertechnetate exposure, scintigraphic analyses (i.e., planar scintigraphy as well as single-photon emission computed tomography/computed tomography (SPECT/CT)) in supine position were performed by means of a double-head gamma camera (Siemens SymbiaT2, Siemens Medical Systems, Hofmann Estates, IL, USA) equipped with low-energy high-resolution collimators. To receive a 360° acquisition, 32 projections with a resolution of 128 × 128 px were recorded with an acquisition time of 8 s per projection. Anterior (i.e., LDR = left–dorsal–right) as well posterior (i.e., RVL = right–ventral–left) planar images were acquired with an acquisition time of 3 min per position. Finally, a low-dose computer tomography (CT) was performed at 130 kV that led to a slice thickness of 5 mm.

Three-dimensional images were iteratively reconstructed based on four iterations and eight subsets (Flash 3D). Afterwards, the images were transferred into image processing software (Siemens Syngo.Via, Siemens Healthcare GmbH, Erlangen, Germany) and analysed by means of suitable add-ons for planar scintigraphy (MM Oncology application) and SPECT-CT (mMR General application).

### Multilocal in-tissue penetration (ITP) depth analyses

Multilocal in-tissue doxorubicin penetration depth analyses were performed after doxorubicin exposure (doxorubicin hydrochloride, Teva^®^, 2 mg/mL, Pharmachemie B.V., Haarlem, Netherlands) of two postmortem pig peritonea by HINAT-LAU and PIPAC-MIP. HINAT-LAU was performed with undiluted doxorubicin (drug chamber was filled with 5 mL) at a system pressure of 3 bar for 25 min that cumulated into an effective doxorubicin dose of 0.88 mL (i.e., 1.76 mg doxorubicin hydrochloride). PIPAC-MIP was performed with diluted doxorubicin (1 mL doxorubicin in 50 mL aqueous 0.9 wt % sodium chloride solution) at a volumetric flow rate of 30 mL/min for 100 s that led to an effective doxorubicin dose of 2.9 mL (i.e., 5.8 mg doxorubicin hydrochloride).

Short after doxorubicin exposure, the pigs underwent median laparotomy during which peritoneal tissue samples with a geometry of 30 × 30 × 5 mm were resected from multiple locations as illustrated in Figure 4, i.e., from the central small bowel beneath the aerosol inlet, the right upper/lower and the left upper/lower as well as from the subdiaphragmal right/left, the epigastric and the posterior stomach. Afterwards, the tissue samples were rinsed with a sterile aqueous 0.9 wt % NaCl solution to eliminate superficial cytostatics, dried with blotting paper and shock frozen in liquid nitrogen.

Analyses of in-tissue doxorubicin penetration depth (i.e., distance between luminal surface and innermost positive staining for doxorubicin accumulation) were performed by confocal laser microscopy (Leica TCS SP8, Leica Microsystems GmbH, Wetzlar, Germany) with immersion oil on ten cryosections (thickness of 7 µm) of each tissue sample which were mounted (VECTASHIELD® H-1200 Mounting Medium with DAPI, Vector Laboratories Inc., Burlingame, USA) prior to stain nuclei.

### Proof of concept in anesthetised pigs (in vivo)

To show that the HINAT-LAU performance is suitable under more realistic conditions, in vivo experiments on anesthetised German Landrace pigs (45–60 kg) were performed at the European Surgical Institute, Norderstedt, Germany after obtaining the authorization of the animal experiment review board of Schleswig-Holstein.

In analogy to the above-described preparation procedure on postmortem pigs, trocar T1 for aerosol access and trocar T2 for video endoscopy were inserted into the capnoperitonea (*p*_C_ = 12 mmHg = 1.6 kPa) of the anesthetised pigs. Furthermore, a third trocar T3 (Kii^®^Balloon Blunt Tip System, 5 mm, Applied Medical, Rancho Santa Margarita, CA, USA) was set to monitor the intra-abdominal temperature by means of a temperature/humidity probe (XA Lufft; Lufft Mess- und Regeltechnik GmbH, Fellbach, Germany). In order to visualise the droplet deposition, HINAT-LAU was operated with an undiluted aqueous methylene blue solution (Toluidinblau; Dr. Franz Köhler Chemie GmbH, Bensheim, Germany) at 3 bar system pressure based on pressurized carbon dioxide. The aqueous methylene blue solution was heated by a thermostat to reach an aerosol temperature of 41 °C. Aerosol charging was performed extracavitary as well as intracavitary by operating a brush electrode (Ionwand^TM^, Alesi Surgical Ltd, Cardiff, UK) of an approved electrostatic precipitation system (Ultravision^TM^, Alesi Surgical Ltd, Cardiff, UK).

## Results und Discussion

### Granulometric analyses

Before discussing the granulometric results, it should be noted here again that the HINAT-LAU was operated with compressed air and that for all granulometric analyses a mean aerosol temperature of 27.8 ± 0.6 °C was maintained. Note that the LAU operation with carbon dioxide would lead to slightly shifted, but similar results. This is due to the simultaneous decrease of both the mass flow rate and the gas flow rate when operating the LAU instead of air with carbon dioxide that is caused by the higher gas density of carbon dioxide (ρ_CO2_(28 °C) = 1.77 kg/m^3^) in comparison to air (ρ_air_(28 °C) = 1.16 kg/m^3^). Theoretical as well as experimental analyses (not presented here) based on LAU operation have shown a good proportionality of both the volumetric flow rate and the mass flow rate to the square root of the gas density ratio, or in other notation for the present consideration, (ρ_air_(28 °C)/ρ_CO2_(28 °C))^0.5^ = 0.81.

**Figure 5:**
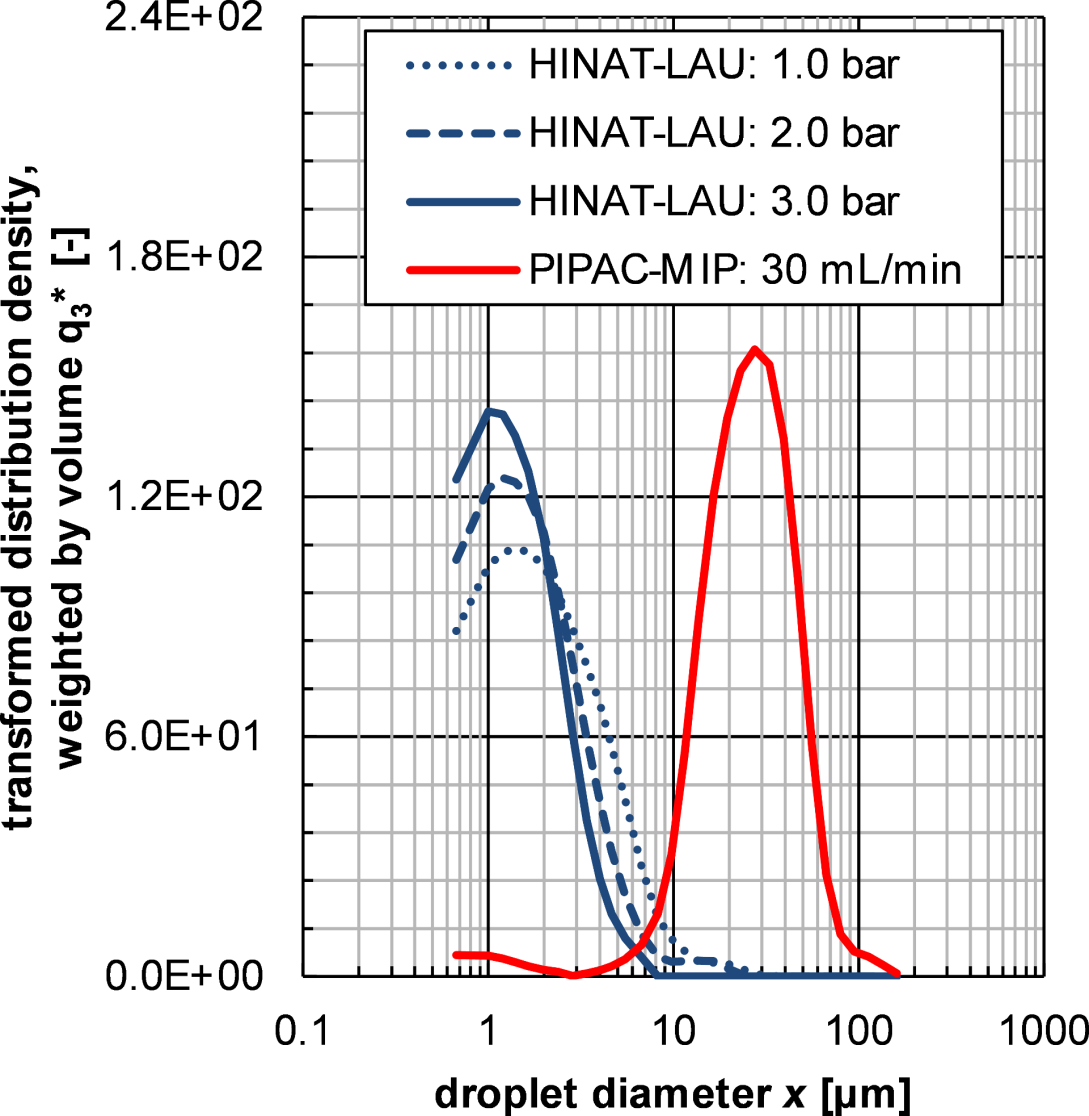
Granulometric results based on laser diffraction spectrometry: Volume-weighted droplet size distributions of the aerosols generated by HINAT-LAU in comparison to the PIPAC-MIP results given in [13].

Figure 5 shows the volume-weighted droplet size distributions (determined by LDS) of the aerosols generated by HINAT-LAU in comparison to PIPAC-MIP as given in [13]. The size distributions in Figure 5 show that the droplet size of the HINAT-LAU aerosol decreases with increasing system pressure, i.e., the volume-weighted modal value of the transformed density function shifts from 1.4 µm at 1.0 bar via 1.2 µm at 2.0 bar to 1.0 µm at 3.0 bar. In contrast to the volume-weighted droplet size distribution of the PIPAC-MIP aerosol, the HINAT-LAU results show either no (i.e., at a system pressure of 3 bar) or only a minor volume-weighted content of droplets >10 µm.

**Figure 6:**
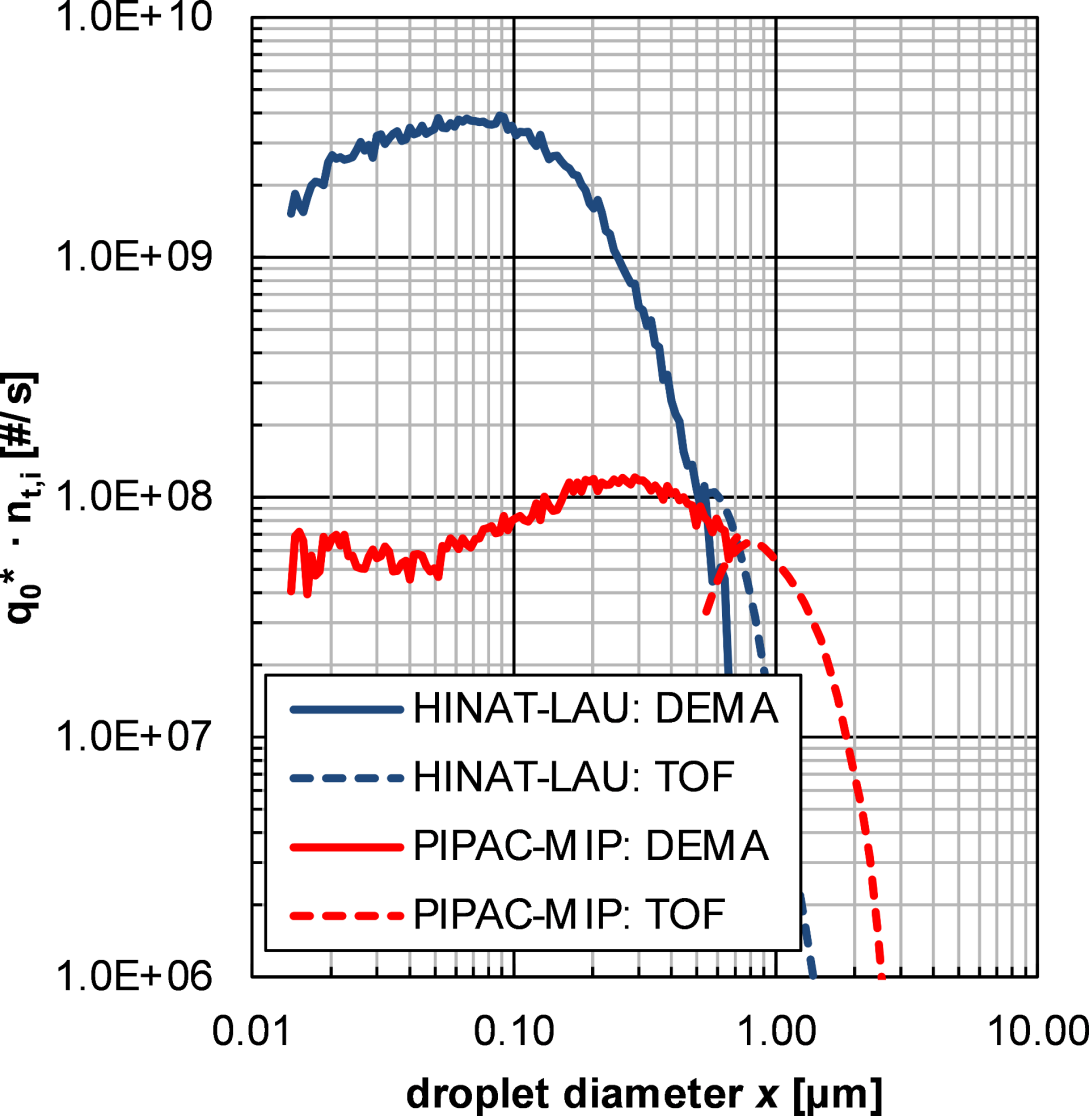
Granulometric results based on differential electrical mobility analyses (DEMA) and time-of-flight spectrometry (TOF): Mean, non-normalised, transformed, number-weighted droplet size distributions of the aerosols generated by HINAT-LAU and PIPAC-MIP.

As mentioned above, DEMA and TOF analyses were performed in order to obtain even more detailed information as well as quantitative data on the fine droplet fraction, especially for the submicrometre size range. The results of these analyses are given in Figure 6 as non-normalised (i.e., scaled by droplet generation rate *n*_t,i_), transformed, number-weighted size distributions for the aerosols generated by HINAT-LAU (1.5 bar system pressure) and PIPAC-MIP (30 mL/min liquid flow rate).

Figure 6 shows that the non-normalised size distributions determined by TOF measurements supplement well the ones of DEMA due to the spherical shape and unit density of the generated droplets. The HINAT-LAU aerosol is characterised by a number-weighted median diameter of *x*_50,0,HINAT-LAU_ = 63.4 ± 1.8 nm and a geometric standard deviation of GSD_HINAT-LAU_ = 2.57 ± 0.07, where the values for the PIPAC-MIP aerosol were *x*_50,0,PIPAC-MIP_ = 254. ± 2.9 nm and GSD_PIPAC-MIP_ = 2.55 ± 0.05.

The non-normalised size distributions given in Figure 6 reveal that the HINAT-LAU aerosol is composed of even more finer droplets than the PIPAC-MIP aerosol despite the 750-fold lower liquid dose rate. In detail, a total droplet generation rate (evaluated according to [19]) of *n*_t,CPC,HINAT-LAU_ = 3.32·10^9^ #/s was determined for HINAT-LAU, which was 25 times higher than that of *n*_t,CPC,PIPAC-MIP_ = 1.35·10^8^ #/s as determined for PIPAC-MIP.

### Spatial distribution of drug deposition

#### Peritoneal drug deposition efficiency for drug dose determination

In contrast to conventional PIPAC, the peritoneal drug deposition efficiency of the HINAT approach, i.e., the ratio between deposited drug mass to the supplied drug mass, depends essentially on the aerodynamic conditions (e.g., residence time, degree of turbulence) caused by the capnoperitoneal volume/geometry and the aerosol flow characteristics, but also on electrostatic and thermophoretic effects. In order to estimate the actual drug dose for scintigraphic peritoneography and multilocal in-tissue penetration depth analyses, the peritoneal drug deposition efficiency was determined gravimetrically by weighing the drug chamber as well the exhaust HEPA filter before and after HINAT-LAU operation. For the given postmortem pig capnoperitonea with a capnoperitonal volume of about 2 L, a peritoneal deposition efficiency of 86 wt % was determined for 1 bar HINAT-LAU system pressure and 47 wt % for 3 bar. A linear interpolation between the given values was used for the estimation of the peritoneal drug deposition at other operation parameters.

#### Scintigraphic peritoneography (SPG)

**Figure 7:**
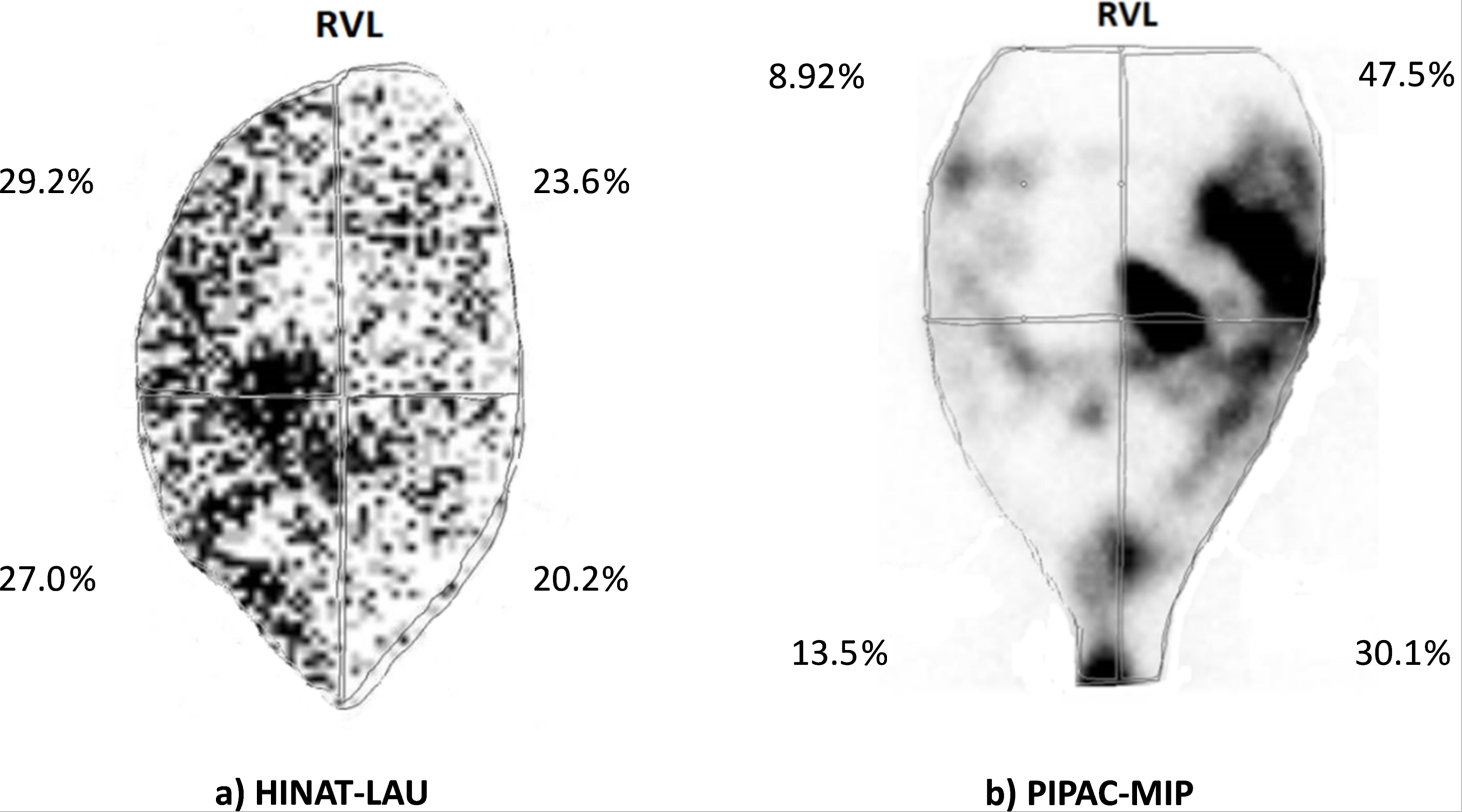
Planar ^99m^Tc pertechnetate distribution within the pig peritoneum after application of a) HINAT-LAU (planar scintigram based on 5757 nuclide counts) and b) PIPAC-MIP (planar scintigram based on 415000 nuclide counts (mod. from [14])); RVL = right–ventral–left.

**Figure 8:**
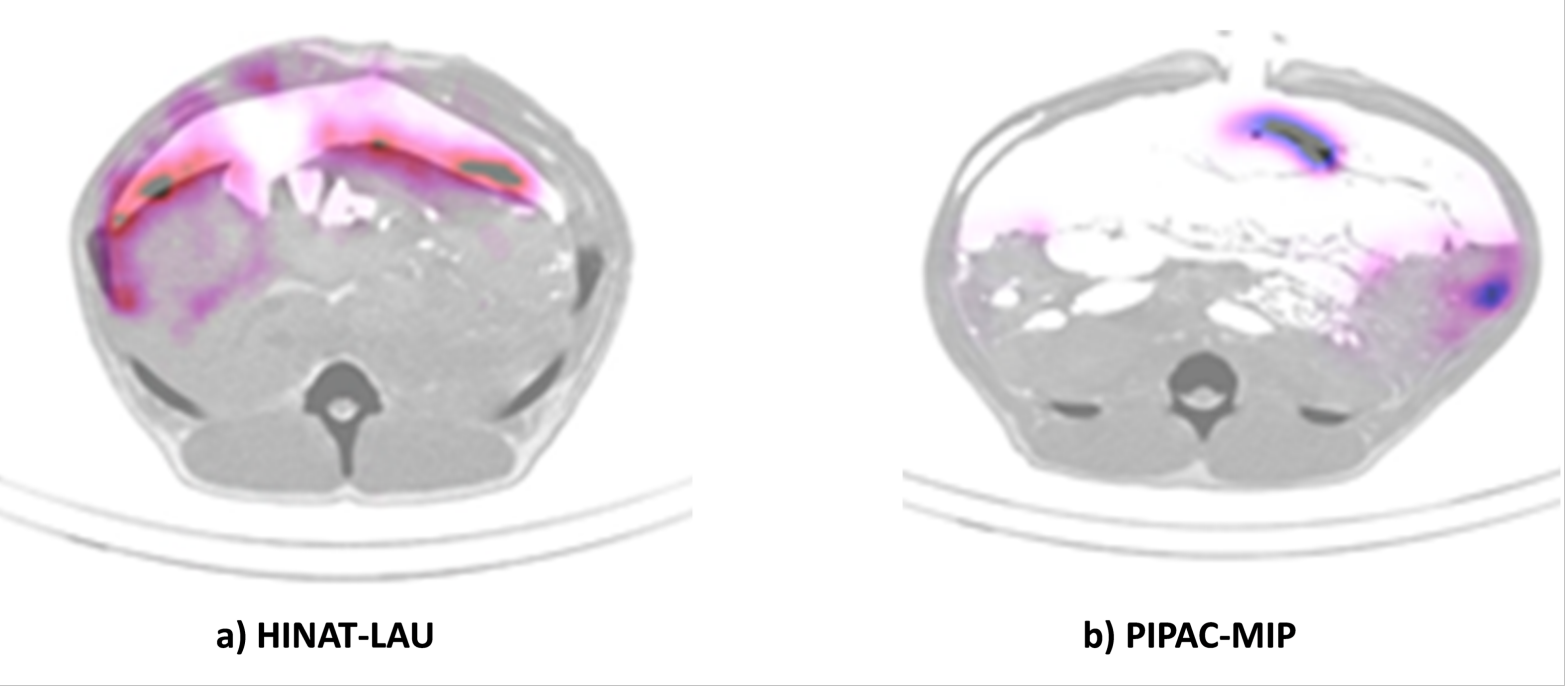
Single-photon emission computed tomography/computed tomography (SPECT/CT) images of ^99m^Tc pertechnetate distribution within the pig peritoneum after application by a) HINAT-LAU and b) PIPAC-MIP.

The ^99m^Tc pertechnetate distribution within the pig peritonea after application by HINAT-LAU and PIPAC-MIP is visualised in Figure 7 and Figure 8.

Both the planar scintigram in Figure 7a and the SPECT/CT image in Figure 8a show that the application by HINAT-LAU led to a quasi-uniform ^99m^Tc pertechnetate distribution over the whole pig peritoneum with a slight increased ^99m^Tc pertechnetate deposition on the visceral peritoneum beneath the aerosol inlet. In contrast to HINAT-LAU, the ^99m^Tc pertechnetate deposition after application by PIPAC-MIP led to hotspots on the peritoneum beneath the MIP and on the paracolic gutter to the cul-de-sac. The relative ^99m^Tc pertechnetate count content within the four quadrants varied between 9% and 48%. These hotspots were found in only 3 vol % of the abdominal cavity but contain about 30% of the delivered ^99m^Tc pertechnetate activity, as determined by SPECT/CT.

#### Multilocal in-tissue penetration depth analysis

**Figure 9:**
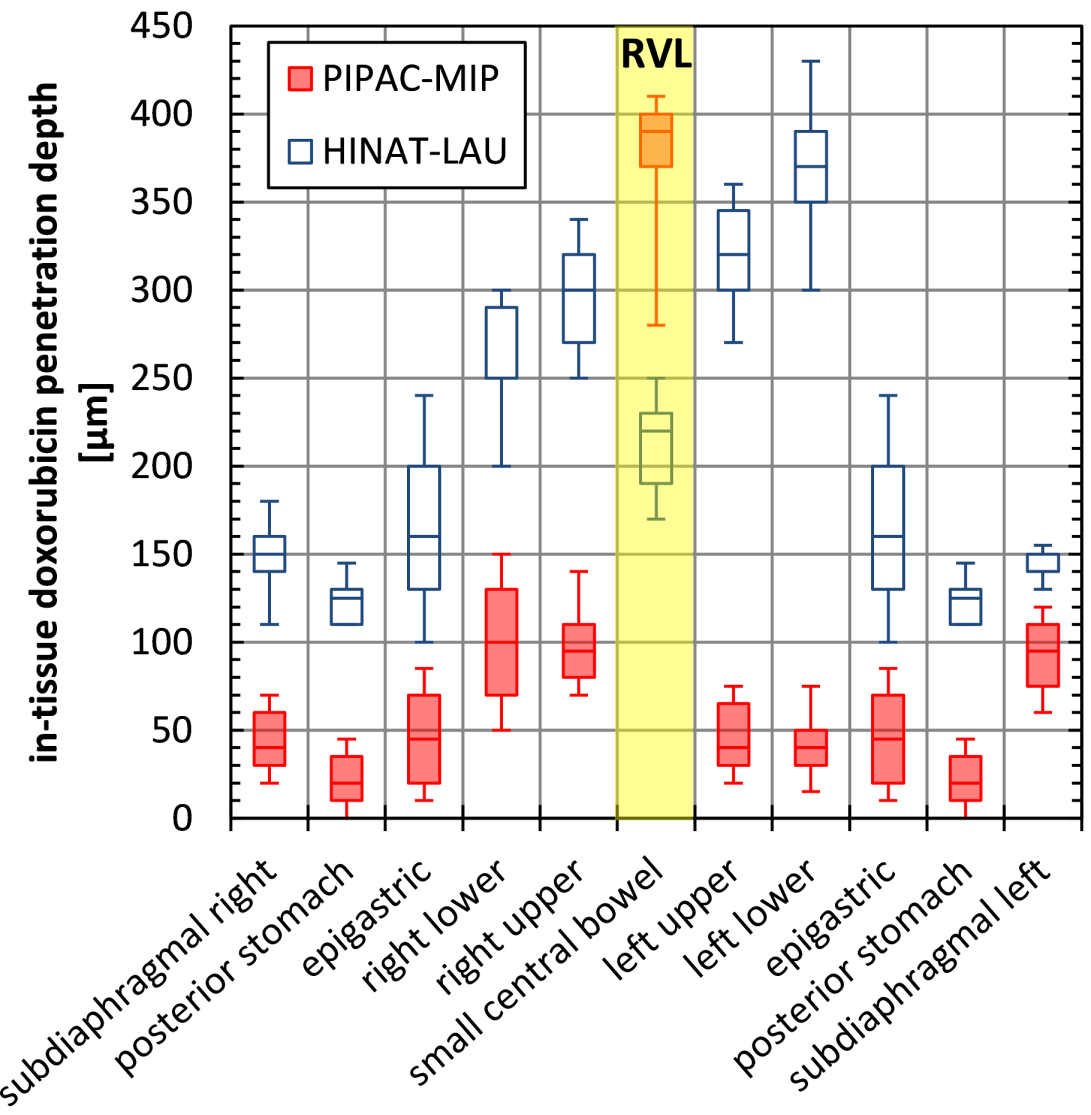
Doxorubicin in-tissue penetration at different regions within pig peritonea after drug application by HINAT-LAU (deposited doxorubicin dose of 0.88 mL) and PIPAC-MIP (deposited doxorubicin dose of 2.9 mL); values for epigastric and posterior stomach are shown twice.

Figure 9 shows the determined doxorubicin in-tissue penetration depth after HINAT-LAU (deposited doxorubicin dose of 0.88 mL) and PIPAC-MIP (deposited doxorubicin dose of 2.9 mL) application.

Except for the penetration depth at the small central bowel, HINAT-LAU shows significantly higher doxorubicin penetration depths at all analysed locations in comparison to PIPAC-MIP, despite the 3-times lower doxorubicin deposition dose. The higher penetration value at the small central bowel based on PIPAC-MIP is attributed to the high volumetric content of micrometre sized droplets deposited due to inertial impaction beneath the MIP nozzle outlet as discussed in [13].

Although the penetration depths based on HINAT-LAU varied less than those of PIPAC-MIP, significant differences can be observed over the sampling locations. This appears contrary to the prior presented scintigraphic results, but can be attributed to the varying drug affinity based on histological and functional differences of the resected tissue samples (e.g., [20–21]).

However, based on the realised experimental conditions, an overall mean penetration depth of 226 ± 88 µm could be determined for HINAT-LAU and 102 ± 104 µm (or 68 ± 38 µm when neglecting the values for the small central bowel) for PIPAC-MIP. Thus, the mean penetration depth of the 3-times lower doxorubicin deposition dose based on the HINAT-LAU aerosol was at least 2-times higher than for the PIPAC-MIP aerosol, which is attributed to the higher concentration gradient of the supplied drug [5,22].

### Proof of concept testing in anesthetised pigs

For the realised two-port operation mode (i.e., aerosol supply/exhaust via trocar T1 and video control via trocar T2), a constant capnoperitoneal carbon dioxide pressure of *p*_C_ = 12 mmHg = 1.6 kPa could be realised by a HINAT-LAU system pressure of ≈3 bar. Therefore, only a minimal pressure regulation was necessary at the beginning. The desired aerosol temperature of 41 °C could be achieved in less than 2 min. The monitoring of the capnoperitonal pressure and aerosol temperature revealed that these values were constant over the whole experimental time for about one hour.

**Figure 10:**
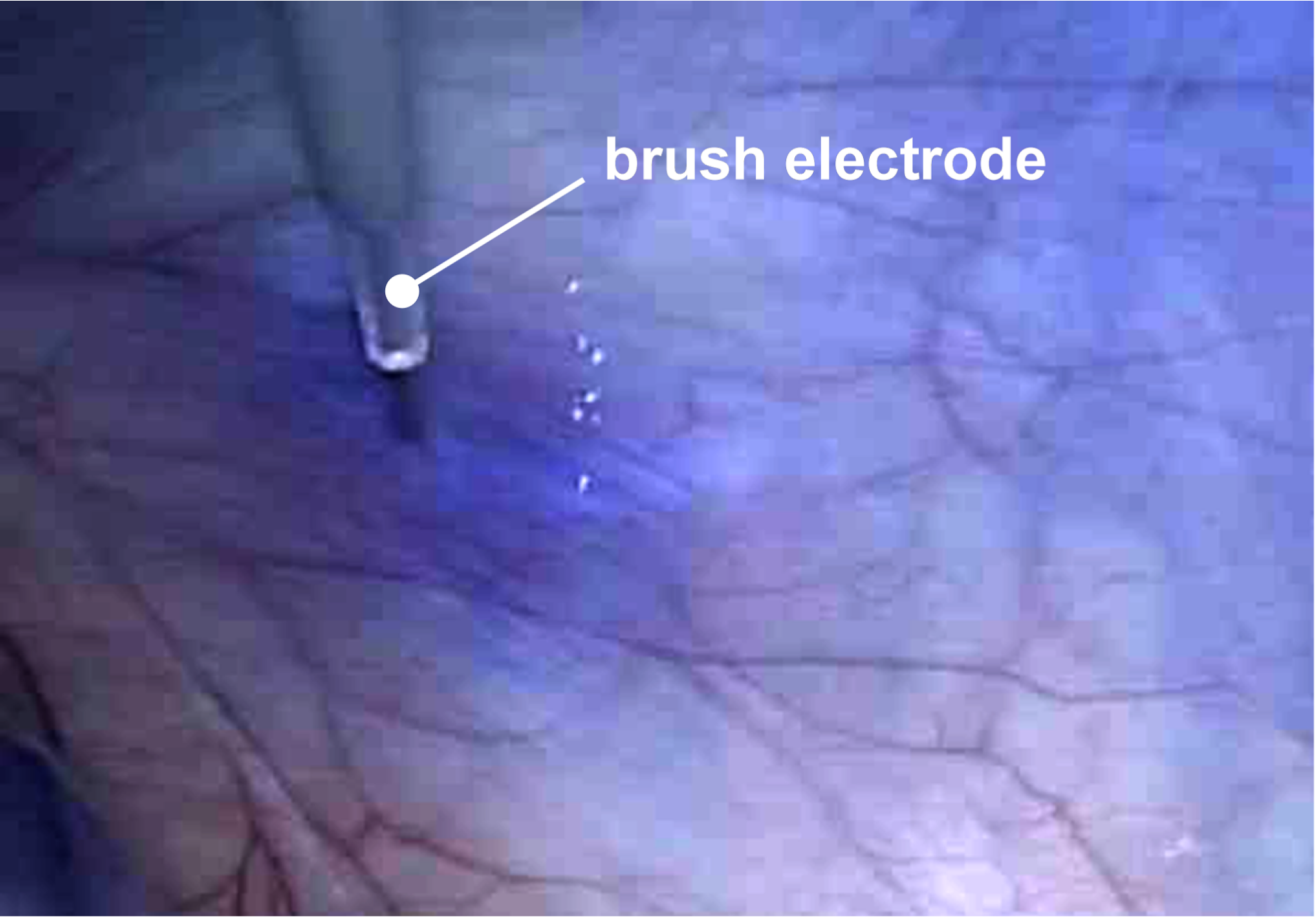
Endoscopic image of the peritoneum exposed to the HINAT aerosol with increased local drug deposition (i.e., blue dyed area) due to electrostatic precipitation based on intracavitary aerosol charging.

The experiments were performed with extracavitary as well as intracavitary aerosol charging. As expected, the even more homogeneously deposited ultrafine droplets of the extracavitary-charged HINAT-LAU aerosol showed neither a local methylene blue staining nor a visible staining of the whole peritoneum. In the case of intracavitary aerosol change, which goes along with an increased droplet deposition based on the formation of an electrical field between electrode and grounded peritoneum, a significant dye staining could be observed near the electrode as shown in Figure 10.

**Figure 11:**
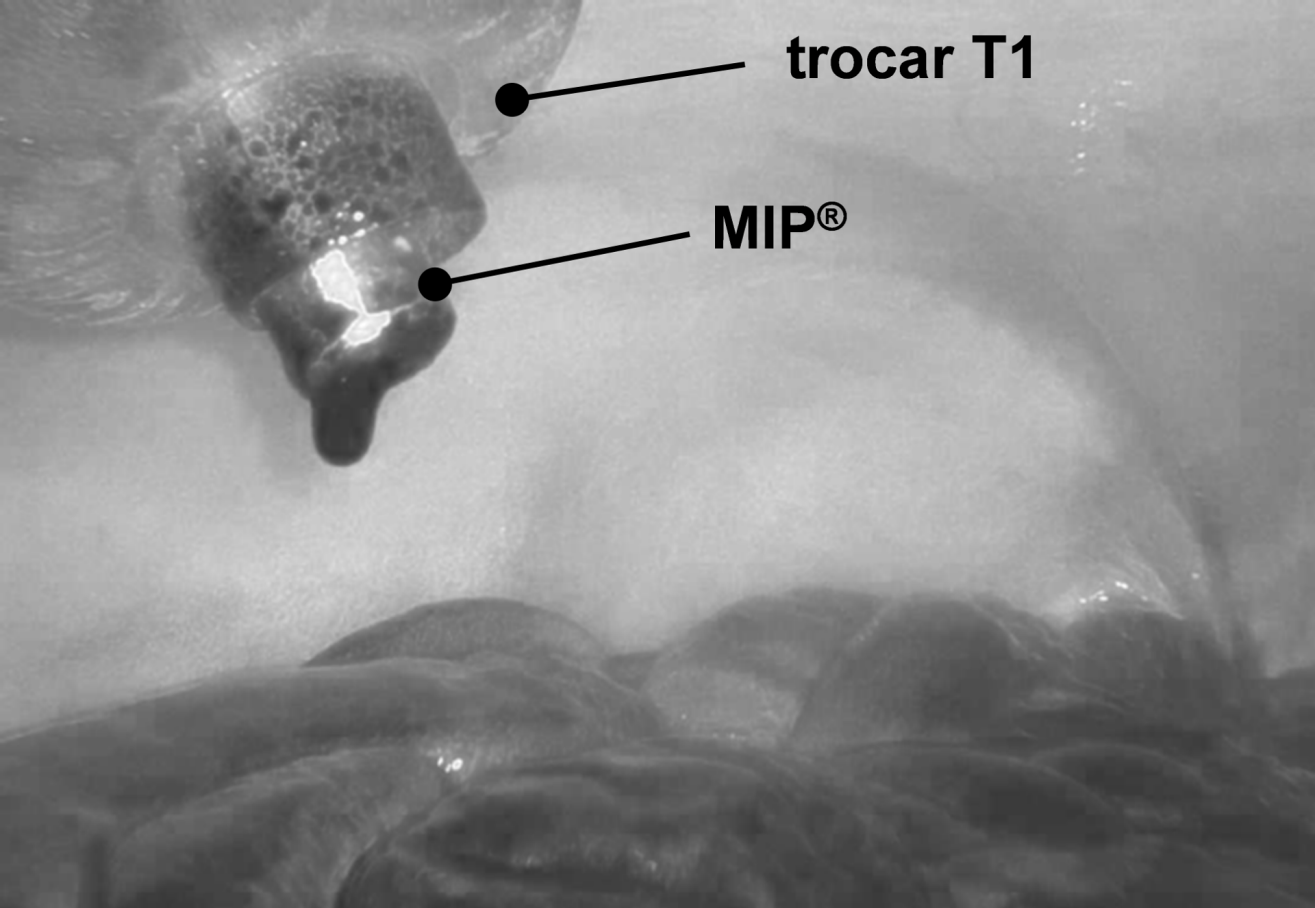
Endoscopic image of the peritoneum after exposure to the PIPAC-MIP aerosol with local drug deposition (i.e., black dyed area) beneath the nozzle outlet.

In contrast to the extracavitary-charged HINAT-LAU aerosol, PIPAC-MIP operation with aqueous methylene blue solution led to an intense dyed area beneath the MIP nozzle outlet as shown in Figure 11.

Last but not least, from the view of the senior surgeon, it is concluded that the HINAT approach benefits also from quick implementation and simple handling at comparably low physical space requirements.

## Conclusion

In order to improve the application of chemotherapeutic drugs for the treatment of peritoneal carcinomatosis during pressurised intraperitoneal aerosol chemotherapy (PIPAC), a novel approach was developed. This approach is based on the extracavitary generation of a hyperthermic and unipolar-charged aerosol composed of nanometre-sized drug droplets for the purpose of capnoperitonal application and is thus called “hyperthermic intracavitary nanoaerosol therapy (HINAT)”.

A comprehensive testing program was realised, wherein both the HINAT approach and conventional PIPAC (designated as PIPAC-MIP) were analysed in parallel. Ex vivo granulometric analyses were performed by laser diffraction spectrometry (LDS), differential electrical mobility analyses (DEMA), time of flight spectrometry (TOF) and condensation nuclei counting (CNC) to characterise the provided aerosols ranging from the nanometre size range up to the micrometre size range. The impact of the different aerosols on the local drug deposition and in-tissue penetration was examined in postmortem pigs by scintigraphic peritoneography (SPG) and multilocal in-tissue penetration depth analyses (ITP). Finally, a fist proof of concept was performed by in vivo analyses in anesthetised German Landrace pigs. The main results of the performed testing program are summarized in Table 1 for both the HINAT approach and PIPAC-MIP.

**Table 1:** Performance parameters and aerosol characteristics of HINAT and PIPAC-MIP as well as experimental results concerning drug deposition homogeneity and in-tissue drug penetration; last two columns provide parameter ratios.

	parameter	units	HINAT	PIPAC-MIP	PIPAC-MIP/ HINAT	HINAT / PIPAC-MIP

general details	nozzle type	[–]	two component	one component	–	–
	nozzle diameter	[mm]	0.5	0.2	0.4	2.5
	system pressure	[bar]	1.0–3.0	6.0–13.8	6 … 4.6	0.17 … 0.22
	volumetric flow rate	[L/h]	210–500	n.d.	–	–
	liquid drug flow rate	[g/h]	4.25–4.5	1250–3000	294–667	0.003–0.002

granulometry (ex vivo)	system pressure	[bar]	1.5	7.3	4.87	0.21
	shape of size distribution	[–]	monomodal	bimodal	–	–
	*n*_t_,_CNC_(< 10 µm)^a^	[#/s]	3.32 × 10^9^	1.35 × 10^8^	0.04	24.59
	*x*_50,3,LDS_^b^	[µm]	≈1.3	25	23.08	0.04
	*x*_50,0,DEMA-TOF_^c^	[nm]	63 ± 2	255 ± 3	4.05	0.25

SPG (postmortem pig)	system pressure	[bar]	1.4	7.3	5.21	0.19
	volumetric flow rate	[L/h]	268	1.8	0.01	148.89
	generation time	[min]	5	5	1.00	1.00
	99^m^ Tc pertechnate dose	[Mbq]	0.28	150	535.71	0.002
	deposition homogeneity	[–]	uniform	local hot spots	–	–

ITP (postmortem pig)	system pressure	[bar]	3	7.3	5.21	0.41
	volumetric flow rate	[L/h]	500	1.8	0.01	277.78
	generation time	[min]	25	1.67	0.33	15.00
	supplied doxorubicin dose	[mL]	0.88	2.9	3.30	0.30
	mean penetration depth	[µm]	226 ± 88	102 ± 104	0.45	2.22

^a^Droplet generation rate determined by CNC, ^b^volume-weighted median droplet size determined by LDS, and ^c^number-weighted median droplet size determined by DEMA-TOF.

The HINAT aerosol showed a monomodal size distribution with a volume-weighted median diameter of around 1.3 µm or a number-weighted median diameter of 63 nm. This is a significant contrast to the bimodal size distribution offered by PIPAC-MIP for which a volume-weighted median droplet size of 25 µm was already observed by Göhler et al. [13]. The number-weighted median diameter of the fine PIPAC-MIP droplet fraction (i.e., <2.5 vol %) was determined to be 255 nm. Despite a several hundred times lower liquid drug flow offered by HINAT, a 25 times higher droplet generation rate was determined as compared to PIPAC-MIP. SPG analysis revealed a quasi-uniform droplet deposition over the whole postmortem pig peritoneum for HINAT, while ITP analyses showed a significantly deeper drug penetration with less local variation. The first proof of concept was successfully performed in anesthetised pigs and showed a simple implementation and handling of HINAT.

Beside the above-mentioned and experimentally verified benefits with respect to PIPAC-MIP, HINAT provides the opportunity to perform further studies concerning the treatment of peritoneal carcinomatosis or other aerosol-accessible kinds of cancer (e.g., lung cancer) on animals even smaller than pigs due to the minimal required access space for feeding nanometre-sized aerosols into body cavities. Furthermore, the liquid atomisation unit of the HINAT approach was designed for multiple use with regard to economic aspects.
